# Elevated Vulnerability of Chronic Leukemia Patients to COVID-19 Infection: A Systems Biology Approach

**DOI:** 10.1007/s44229-022-00005-y

**Published:** 2022-05-13

**Authors:** Abdulkhaliq J. Alsalman, Mohammed Al Mohaini, Md. Zubbair Malik, Mohd. Imran, Fadhel A. Alomar, Nasir Al Awwad

**Affiliations:** 1 Department of Clinical Pharmacy, Faculty of Pharmacy, Northern Border University, Rafha, 91911 Saudi Arabia; 2 Basic Sciences Department, College of Applied Medical Sciences, King Saud Bin Abdulaziz University for Health Sciences, Alahsa, Saudi Arabia; 3 King Abdullah International Medical Research Center, Al-Hasa, Saudi Arabia; 4 School of Computational and Integrative Sciences, Jawaharlal Nehru University, New Delhi , 110025 India; 5 Department of Pharmaceutical Chemistry, Faculty of Pharmacy, Northern Border University, Rafha, 91911 Saudi Arabia; 6 Department of Pharmacology and Toxicology, College of Clinical Pharmacy, Imam Abdulrahman Bin Faisal University, Dammam, 31441 Saudi Arabia; 7 Department of Clinical Pharmacy, Faculty of Clinical Pharmacy, Albaha University, Al Bahah, Al Bahah Province Saudi Arabia

**Keywords:** Leukemia disease, SARS-CoV-2, Network medicine, COVID-19, System biology

## Abstract

**Background:**

Emerging evidence has shown that SARS-CoV-2 may affect the circulatory system in addition to the human respiratory system. However, no study has indicated whether patients with leukemia have a greater likelihood of SARS-CoV-2 infection or have poor treatment outcomes.

**Objective:**

The study aimed to demonstrate the relationship between essential blood proteins and the major SARS-CoV-2 proteins by network pharmacology bioinformatics analysis.

**Methods:**

Bioinformatics analysis was used to establish eight differentially expressed gene hubs in leukemia through differential gene screening, protein–protein interaction network analysis, and gene enrichment analysis. Molecular docking analysis was also conducted to dock the two up-regulated proteins with the spike glycoprotein in leukemia and the critical protease enzyme (Mpro) of SARS-CoV-2.

**Results:**

We identified two up-regulated genes (PTPRC and BCL6) among the eight differentially expressed genes. The PTPRC and BCL6 also docked perfectly with the main SARS-CoV-2 structural proteins.

**Conclusion and Recommendation:**

This study indicates that SARS-CoV-2 is likely to affect with the blood in patients with chronic leukemia. Therefore, patients with chronic leukemia require greater medical attention and precautions during the COVID-19 pandemic.

**Supplementary Information:**

The online version contains supplementary material available at 10.1007/s44229-022-00005-y.

## Introduction

Coronaviruses (family Coronaviridae) are viruses whose genomes comprise single-stranded positive-sense RNA ranging from 27 to 34 kb in size [1]. Coronaviruses gained substantial scientific attention in early 2000 after the severe acute respiratory syndrome (SARS-CoV) and Middle East respiratory syndrome (MERS-CoV) epidemics, which caused approximately 700 and 400 deaths, respectively [2]. In early December, the reporting of SARS-CoV-2 in and around Wuhan, China, alarmed scientific communities about a disease known as COVID-19 [3–5]. Patients infected with SARS-CoV-2 show severe respiratory abnormalities and difficulty breathing, which may eventually result in death [6]. A highly contagious mode of transmission and the prolonged stability of the virus in the air and inert surfaces such as steel are major reasons for its spread worldwide [7]. Thus, in the present global emergency of the COVID-19 pandemic, an urgent need exists to develop an efficient treatment against SARS-CoV-2 infection. With its single-strand positive-sense RNA genome and limited structural and functional protein resources, SARS-CoV-2 can infect host cells and proliferate within them [8]. SARS-CoV-2 hijacks the host machinery at the molecular level to complete its life cycle and produce functional virion progeny [9]. Awareness of the process of COVID-19 is scarce but rapidly growing among patients with cancer, particularly hematologic malignancies. The infection rate in patients with cancer may be higher than that in the general population [10, 11]. In two studies in China, only 10 out of 1099 and 18 out of 1590 patients with COVID-19 were diagnosed with cancer [12, 13]. In one study, 60% of patients with COVID-19 with blood cancer recovered from COVID-19 within a 14-day observation period [14].

The scientific community has published findings on COVID-19 in patients with cancer worldwide [15–18]. Patients with leukemia are frequently myelosuppressed, immunosuppressed, and possibly immunoglobulin deficient, thus making them potentially highly vulnerable to COVID-19 [19]. Because of the disease biology of leukemia subtypes, associated therapy, underlying comorbidities, patient-specific aspects, and specific COVID-19-related risk factors, patients with leukemia may be at a significantly greater threat of developing SARS-CoV-2 infection [19]. Unfortunately, because of minimal reports related to leukemia, the implications are poorly understood in this particular population. Because COVID-19 is a new human virus, whether variations exist relative to other blood cancers and how the virus affects people with leukemia remain unknown. Patients with blood cancer, because of their immunocompromise due to both cancer and the effects of cancer treatment, are likely to be particularly prone to SARS-CoV-2 infection.

Our current study aimed to measure the likelihood of patients with leukemia acquiring SARS-CoV-2 infection, on the basis of a theoretical network biology approach. We studied the interaction of target genes/proteins between leukemic chronic lymphocytic and chronic myelogenous tissue and the SARS-CoV-2 virus by using computational techniques such as screening differentially expressed genes (DEGs), gene enrichment analysis (GEA), construction, protein–protein interaction network analysis (PPINA), and molecular docking analysis (MDA).

## Materials and Methods

**Fig. 1:**
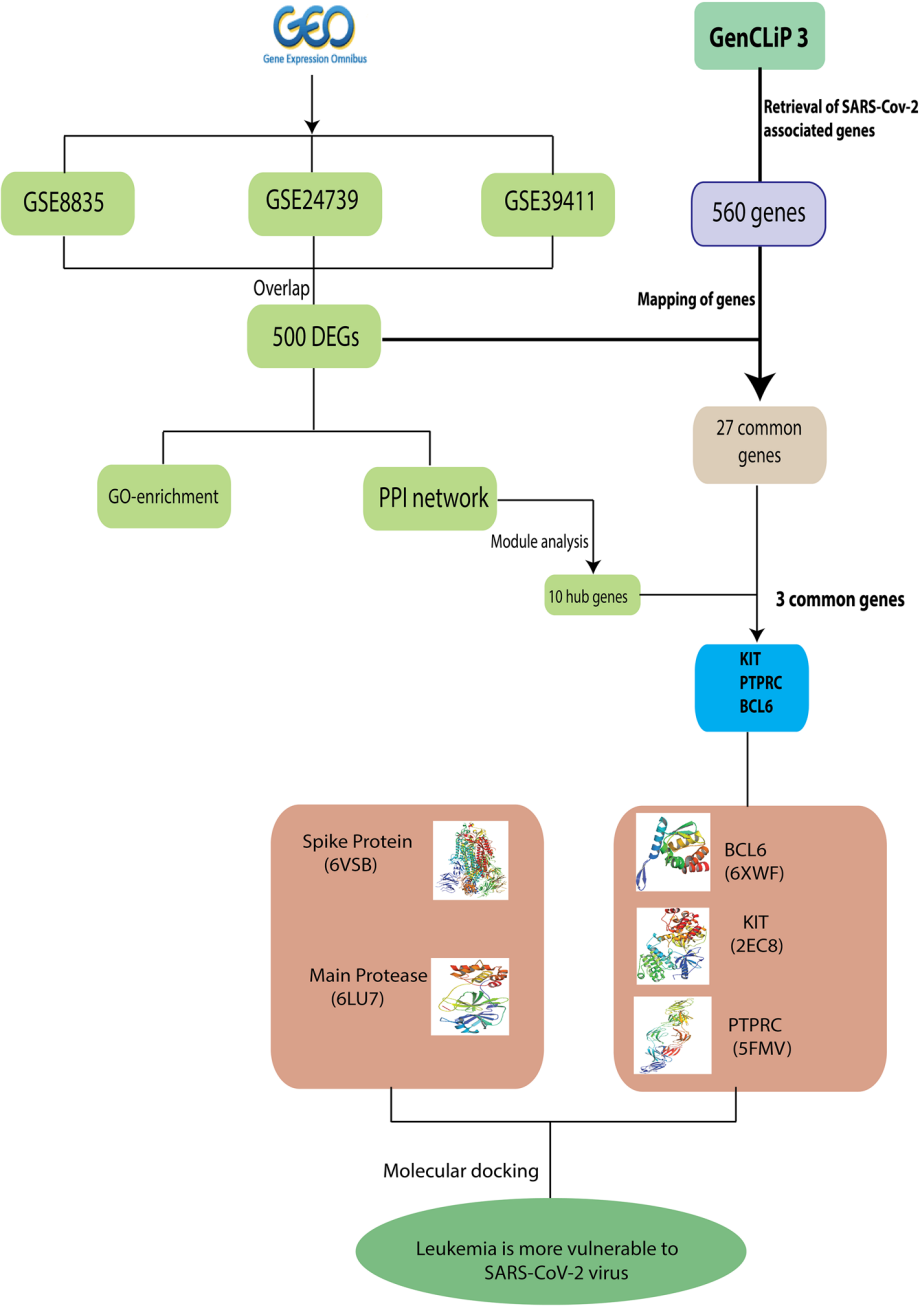
Schematic workflow of the methods implemented in our study

The detailed study workflow is depicted in Fig. 1.

### Microarray Dataset Collection and Preprocessing

Three microarray datasets comprising mRNA expression profiles for leukemia and healthy groups were downloaded from NCBI’s Genome Expression Omnibus (GEO) [20]. The blood datasets included GSE8835 [21], GSE24739 [22], and GSE39411 [23]. These datasets met the following conditions: (1) samples from chronic lymphocytic leukemia (CLL) and chronic myelogenous leukemia (CML) in *Homo sapiens*, (2) presence of control groups, (3) expression profiling by array category, and (4) inclusion of five samples or more. The overall sample size reliably indicates DEGs or non-coding RNAs; therefore, GEO datasets encompassing at least ten samples were selected for further examination. Background data correction/normalization were performed by multiarray average (RMA) in the R affy and Lumi packages to ensure unbiased and dysregulated gene expression data. The RMA approach, including quantile normalization, was used to eliminate variations attributable to the individual Affymetrix GSE series standardization. In the fold-change calculation to identify DEGs, the sensitivity and specificity of the RMA technique were acceptable. We additionally used the Bioconductor Package (Lumi pipeline) designed to study Illumina data (BeadChip). The results verified the consistency, normalization, and stable variance of the data.

### Identification of DEGs

To examine DEGs in every GEO dataset, we used the linear model for microarray analysis (limma) package in R. By using the empirical Bayes method and decreasing the standard errors, it calculates simple *t* test, moderate *t* test, and *f* test results, and offers reproducible results. The limma package [24] was used to determine the DEGs between healthy and leukemia groups. DEGs were characterized as genes with *p* < 0.05, and logFC (0.2 ≥ logFC ≤ ** − **0.2) for up- and down-regulated DEGs, respectively.

### Meta-Analysis of DEGs in the Gene Expression Dataset

Using the MetaMa package [25] and limma [26] in R, we performed meta-analysis of the normalized gene expression datasets by using Fisher's combined probability test technique [27]. False discovery rate adjustment was performed with Benjamini–Hochberg correction [28] by combining the *p*-values and fold-change values of the shared genes. Meta-analysis of datasets was conducted through generic methods of combining information by vote-counting (Table 1). BRCW (http:/jura.wi.mit.edu/bioc/tools/compare.php) was used to identify the mutual DEGs in at least two gene expression profile datasets, thus increasing the accuracy of DEG selection; the chances of a biased data compilation thus became nil. The probe numbers in the expression profile were translated to gene symbols with the Synergizer database, on the basis of the equivalent similarity between the probe and the gene in the data [29].

**Table 1:** List of datasets used in the meta-analysis

GEO Accession	Sample size		Platform	Tissue
	Healthy	Leukemia		
GSE8835	24	42	GPL96: Affymetrix Human Genome U133A Array	Chronic lymphocytic
GSE39411	48	104	GPL570: Affymetrix Human Genome U133 Plus 2.0 Array	Chronic lymphocytic
GSE54536	8	16	GPL570: Affymetrix Human Genome U133 Plus 2.0 Array	chronic myelogenous

### Functional and Pathway Enrichment Analyses

We classified DEGs by biological process (BP), molecular function (MF), cellular component, and Kyoto Encyclopedia of Genes and Genomes (KEGG) pathways to understand the significance of the listed DEGs, on the basis of Database for Annotation Visualization and Integrated Discovery (DAVID) v.6.7 [30]. The Gene Ontology (GO) and KEGG databases are used by DAVID for gene enrichment study. Pathways and roles with a *p* value < 0.05 were considered significant. Subsequently, ggplot2 [31] was used to construct an enrichment plot of critical biological processes, molecular, cellular components, and function pathways.

### Construction of a PPI Network

For PPI network construction and analysis, we obtained specific DEGs through enrichment analysis. We used the simple concept of the correspondence of one gene to one protein and developed the DEGs' primary leukemia PPI network. The network was built with the STRING v.10.5 database [32], and the Cytoscape [33] file has been uploaded for further literature verification.

### Overlap Between Leukemia and SARS-CoV-2-Associated Proteins

We identified reported COVID-19 associated genes, which were searched with GenCLiP3 [34]. GenCLiP 2.0 (http://ci.smu.edu.cn/genclip3/) is an online tool used to analyze human genes for literature mining. The literature mining gene retrieval of COVID-19-associated genes was based on user-defined query keywords. The keywords, grouped by a fuzzy algorithm, can be input by users or generated for the relevant gene established on accessible terms in the prior art. Associated Medline abstracts were linked by using the associations between genes and keywords. The co-occurrence of genes and keywords was highlighted in our literature mining.

### Module Analysis

We used the molecular complex detection (MCODE) app in Cytoscape [35] to perform module analysis with the degree cutoff criteria = 2, node density cutoff = 0.1, node score cutoff = 0.2, *k*-core = 2, and maximum depth = 100. We also studied GO and KEGG pathway enrichment for important genes in hub modules (*p* value < 0.05).

### Correlation of Gene Expression with Tumor-Infiltrating Immune Cells

Given the importance of immune dysregulation in leukemia, we explored the correlations between MTG1, PPP2R5B, and ANXA5 mRNA expression and tumor-infiltrating immune cells. The TIMER tool (https://cistrome.shinyapps.io/timer/) [36] was used to analyze the association between gene expression with tumor-infiltrating cells. Six tumor-infiltrating immune subsets, including B cells, CD8+ T cells, CD4+ T-cells, macrophages, neutrophils, and dendritic cells, were analyzed.

### Protein Preparation

The BCL6 (PDB ID: 6XWF), KIT (PDB ID: 2EC8), and PTPRC (PDB ID: 5FMV) crystal structures were from the RCSB Protein Data Bank (PDB). Using RCSB PDB, we also downloaded the recently submitted crystal structures of COVID-19 spike glycoprotein with a single receptor-binding domain and the main protease (Mpro) of COVID-19 in complex with an inhibitor N3. PyMOL was used to optimize the structures, mainly through removal of ligands and water molecules.

#### Molecular Docking

Rigid molecular docking of proteins was performed with the Cluspro 2.0 [37] server. The files were downloaded from the top ten predictions from the Cluspro web server. Prodigy (https://bianca.science.uu.nl/prodigy/) was used to evaluate the effects of protein docking interactions and provide the binding affinity (Δ*G*). The Δ*G* specifies the solvation free energy (kcal/M) expansion after the formation of the interface. The Δ*G* value is computed as the difference in total solvation energies of isolated and interfacing structures. On the basis of the anticipated Δ*G* (Eq. 1), the dissociation constant (*K*_d_) was determined,

1 $$\Delta G = RT \mathrm{ln}K$$

where *R* is the ideal gas constant (in kcal K^**−**1^ mol^**−**1^), *T* is the temperature (in K), *K* is the equilibrium constant, and Δ*G* is the anticipated free energy 298.15 K (25 °C). Negative Δ*G* is associated with hydrophobic interfaces or positive protein affinity. PRODIGY [38] (PROtein binDIng enerGY prediction) is a web server for calculating binding affinity in biological complexes and determining biological interfaces from crystallographic ones. PyMOL [39] was used for the visualization of the docked structure.

## Results

The flowchart of our bioinformatics analysis of network pharmacology is shown in Fig. 1.

### Extraction and Preprocessing of Microarray Data

**Fig. 2:**
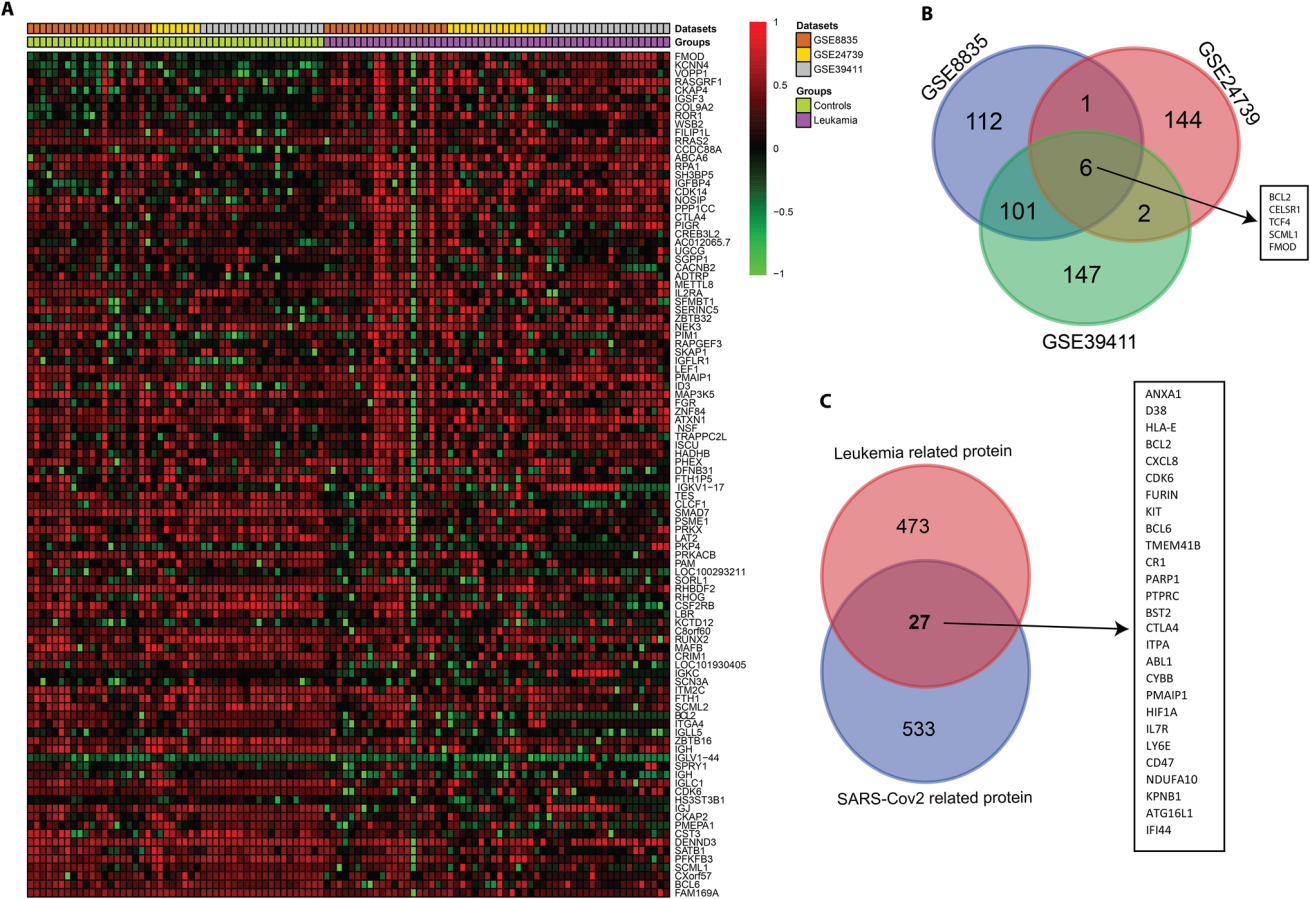
**A** Expression heatmap showing expression levels of significant DEGs for the GSE8835, GSE24739, GSE39411 datasets. Color indicates high expression (red) and low expression (green). **B** Venn diagram showing the DEGs in GSE8835, GSE24739, and GSE39411 after overlap analysis. **C** Venn diagram showing the common genes after mapping of DEGs in leukemia and SARS-CoV-2-associated proteins

Built on the exclusion/inclusion criteria described in the methods, the microarray gene expression profiles with accession numbers GSE8835, GSE24739, and GSE39411 contain expression data from tissues from patients with CLL and CML, and healthy controls. Information associated with these datasets, such as GEO accession number, platform type, number of samples, type of study, and species, is shown in Table 1. The heatmap visualization of expression profiles for tissue samples from patients with CLL and CML and controls is shown in Fig. 2A.

### Meta-analysis and Identification of DEGs in Patients with Leukemia

The three described datasets were used to identify DEGs between patients with leukemia and healthy participants, and to perform meta-analyses for identifying mutually expressed genes across these datasets. A total of 556 DEGs were obtained after meta-analysis, 261 of which were up-regulated and 295 of which were down-regulated in patients with leukemia vs. controls on the basis of the statistical threshold of log2 (fold change) and Benjamini–Hochberg *p*-value.

### Functional and Pathway Enrichment Analysis

**Fig. 3:**
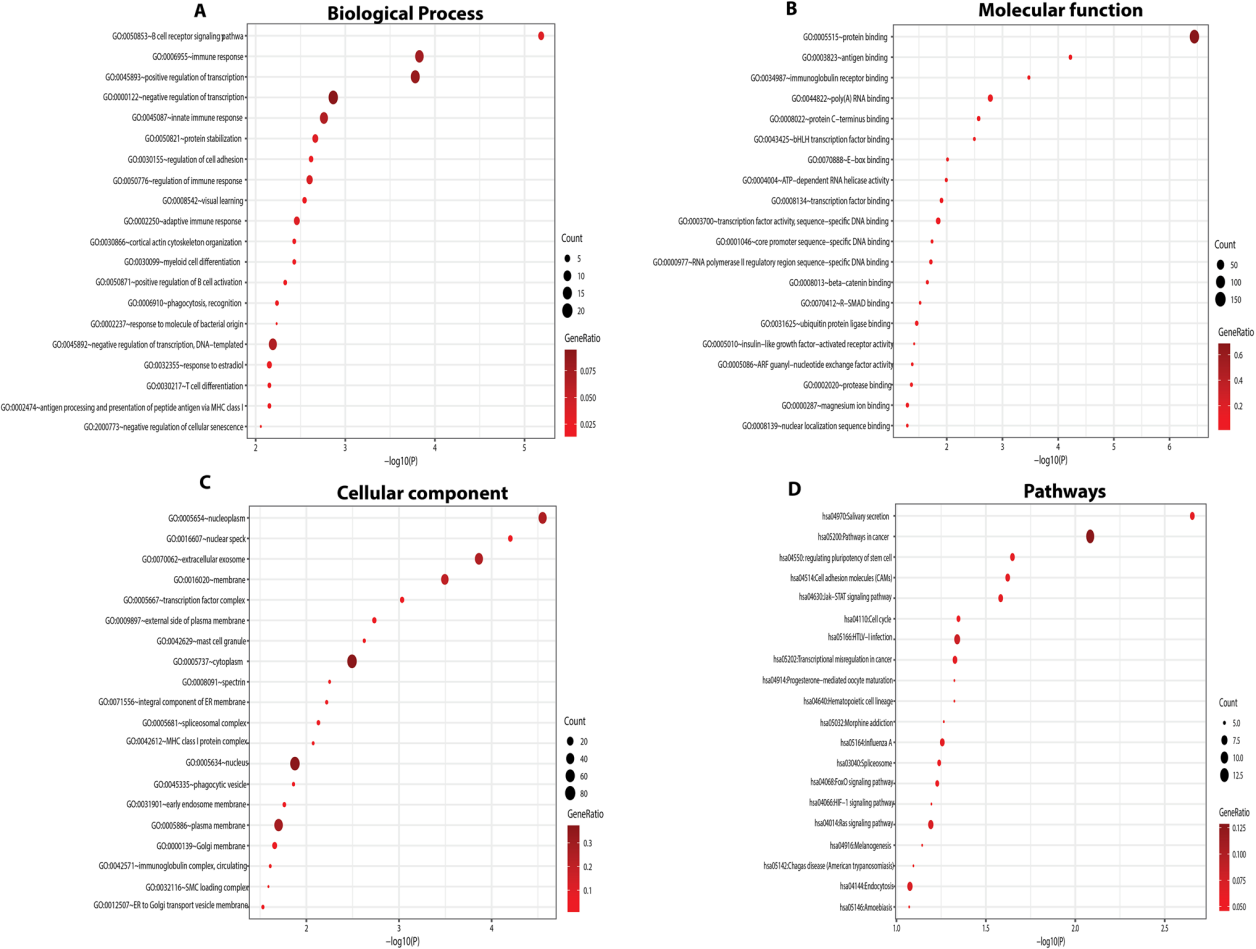
Functional and pathway enrichment analyses of up-regulated DEGs in leukemia (chronic lymphocytic and myelogenous). **A** Biological process enrichment analysis of up-regulated DEGs. **B** Molecular function. **C** Cellular component. **D** KEGG pathway enrichment analysis

**Fig. 4:**
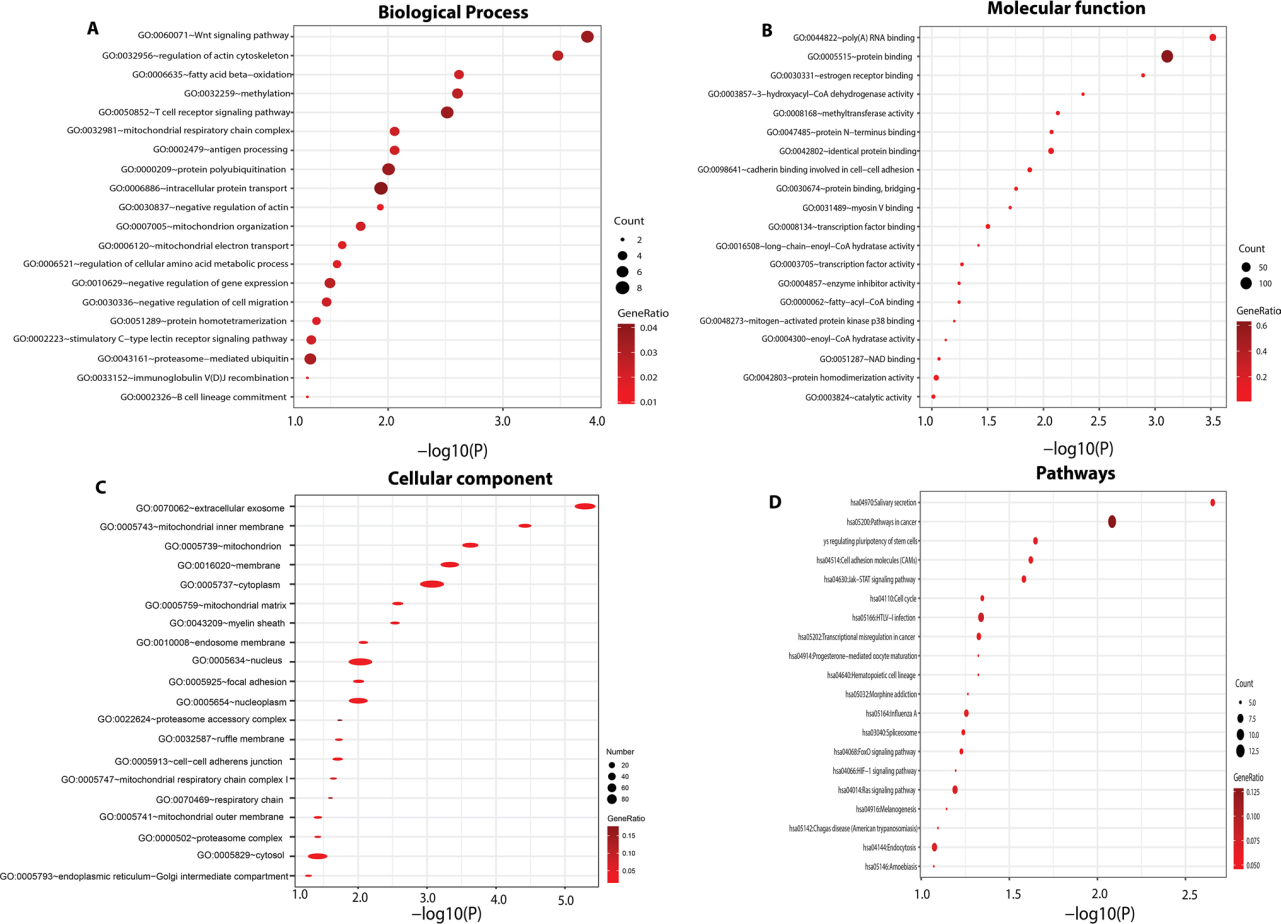
Functional and pathway enrichment analyses of down-regulated DEGs in leukemia (chronic lymphocytic and myelogenous). **A** Biological process enrichment analysis of up-regulated DEGs. **B** Molecular function. **C** Cellular component. **D** KEGG pathway enrichment analysis

We conducted GO enrichment analysis to illustrate the possible biological functions of DEGs in leukemia. GO enrichment analysis and KEGG pathway analysis for the up-and down-regulated DEGs (Supplementary Files 3 and 4) were performed. The significant enrichment of up- and down-regulated DEGs in CLL and CML is shown in Figs. 3 and 4, respectively. The common up-regulated DEGs were enriched in BPs including B-cell receptor signaling pathway, immune response, positive control of transcription, DNA-templated, innate immune response, control of cell adhesion, control of immune response, positive control of B-cell activation, phagocytosis, DNA-templated response to estradiol, T-cell distinction, antigen processing and presentation of peptide antigen via MHC class I, and negative regulation of cellular senescence (Supplementary File 3 and Fig. 3A). Down-regulated DEGs were significantly enriched in BPs including the Wnt signaling pathway, the planar cell polarity pathway, the T-cell receptor signaling pathway, mitochondrial respiratory chain complex I assembly, antigen processing and presentation of exogenous peptide antigen via MHC class II, intracellular protein transport, control of the cellular amino acid metabolic process, negative control of gene expression, negative control of cell migration, protein heteromerization, B-cell lineage commitment, immunoglobulin V(D)J recombination, etc. (Supplementary File 4 and Fig. 4A). Additionally, the MFs of the up-regulated DEGs in blood were protein binding, antigen binding, immunoglobulin receptor binding, protease binding, R-SMAD binding, etc. (Supplementary File 3 and Fig. 3B). Furthermore, the MFs of down-regulated DEGs in the blood were poly(A) RNA binding, protein binding, cadherin binding implicated in cell–cell adhesion, myosin V binding, NAD binding, catalytic activity, etc. (Supplementary File 4 and Fig. 4B). The significant cellular components of up-regulated DEGs included nucleoplasm, extracellular exosome, mast cell granule, spliceosomal complex, MHC class I protein complex, phagocytic vesicle, immunoglobulin complex, circulating, viral nucleocapsid, Golgi apparatus, crucial elements of the membrane, etc. (Supplementary File 3, Fig. 3C). The significant cellular components of down-regulated DEGs included proteasome accessory complex, respiratory chain, mitochondrial respiratory chain complex I, endosome membrane, extracellular exosome, etc. (Supplementary File 4, Fig. 4C). The significant KEGG pathways in up-regulated DEGs included salivary secretion, pathways in cancer (i.e., Wnt-pathways, GSK3 pathways, and FGF signaling pathways), signaling pathways controlling pluripotency of stem cells, cell adhesion molecules, the Jak-STAT signaling pathway, HTLV-I infection, Influenza A, the FoxO signaling pathway, the HIF-1 signaling pathway, the Ras signaling pathway, melanogenesis, endocytosis, etc. (Supplementary File 3, Fig. 3D). Most down-regulated DEGs were involved in KEGG pathways involving the blood, including biosynthesis of antibiotics, Fc gamma R-mediated phagocytosis, metabolic pathways, the Wnt signaling pathway, the Hippo signaling pathway, proteoglycans in cancer, etc., as shown in Fig. 4D (Supplementary File 4).

### PPINA

**Fig. 5:**
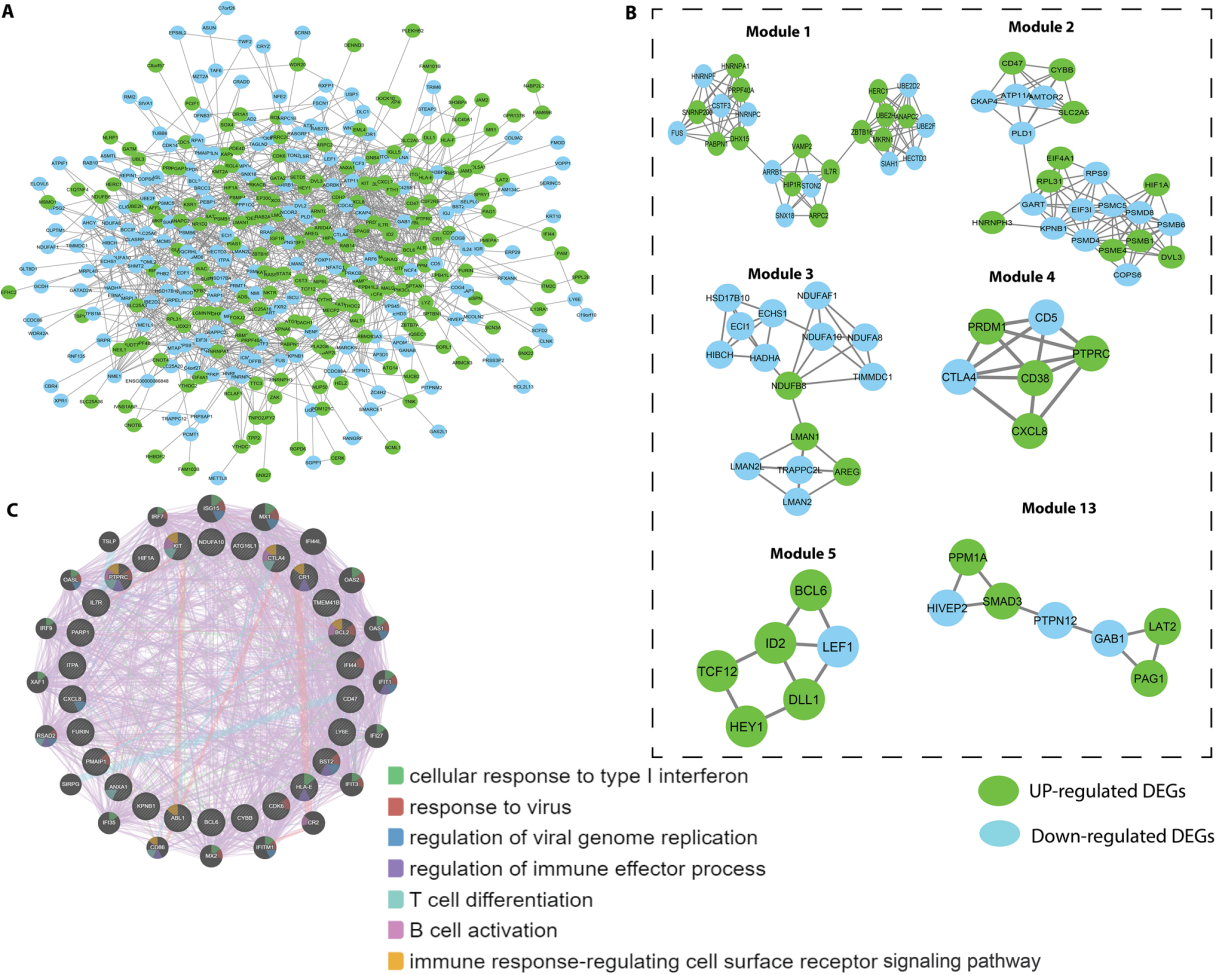
Protein–protein interaction (PPI) network and hub clustering modules. **A** The PPI network of overlapping DEGs. Green nodes represent up-regulated DEGs, and cyan nodes represented down-regulated DEGs. **B** Module 1 (MCODE score = 7.917), Module 2 (MCODE score 7.818), Module 3 (MCODE score = 5.2). Module 5 (MCODE score = 3.2). Module 6 (MCODE score = 3.2) and Module 13 (MCODE score = 2.667)

Using the STRING v.10.5 databases, we built PPI networks and visualized and analyzed them in Cytoscape software v.3.4.0. The PPI evidence acquired from STRING's online DEGs and the PPI network is shown in in Fig. 5A. Network Analyzer, a Cytoscape plugin, was used to analyze the network topological properties (Supplementary File 6: Table S10) of DEGs. The 500 genes showed a considerable degree distribution, with the highest degree of 41 and lowest degree of 1.0. The average degree value was 7.029. The PPI network consisted of 385 nodes and 1353 interactions (Fig. 5A). Finally, the top ten high-grade hub genes, including CDC42, EP300, KIT, PTPRC, DVL2, BCL6, GART, HNRNPA1, HNRNPC, and SMAD3, were selected.

### Overlap Between Leukemia and SARS-CoV-2-Associated Proteins

The overlap between the 500 leukemia DEGs and SARS-CoV-2-associated proteins (560 proteins) was analyzed with Venn diagrams (Fig. 2C). A total of 27 leukemia-regulated SARS-CoV-2-associated proteins were identified, including ANXA1, CD38, HLA-E, BCL2, CXCL8, CDK6, FURIN, KIT, BCL6, TMEM41B, CR1, PARP1, PTPRC, BST2, CTLA4, ITPA, ABL1, CYBB, PMAIP1, HIF1A, IL7R, LY6E, CD47, NDUFA10, KPNB1, ATG16L1, and IFI44. Of the ten hub genes, three hubs, KIT, PTPRC, and BCL6, were common between leukemia and SARS-CoV-2-associated proteins. PPI network constructs with 27 genes commonly involved in leukemia and COVID-19 are shown in Fig. 5C. According to GO and KEGG pathway enrichment examination, most of the genes were implicated in multiple responses, including response to interferon, cellular response to type I interferon, viral response, control of viral genome replication, control of immune effector process, T-cell differentiation, B-cell activation, and immune response-controlling cell surface receptor signaling pathways.

### Potential Mechanisms of the Critical Genes

The TIMER web tool [36] indicated that expression of the KIT, PTPRC, and BCL6 genes was meaningfully associated with one or more blood cancer (lymphoma)-infiltrating immune cell subsets. For B-cells, the expression of PTPRC and BCL6 displayed the most meaningful connection, and the expression of CDC42 was the most important relationship. CD4+ T cells have been demonstrated to support B cells to produce antibodies and help CD8+ T cells eradicate cells infected with SARS-CoV-2 viruses. Interferon-gamma, the leading player governing viral infection, is a major cytokine made by T cells [40].

### Network module analysis

**Fig. 6:**
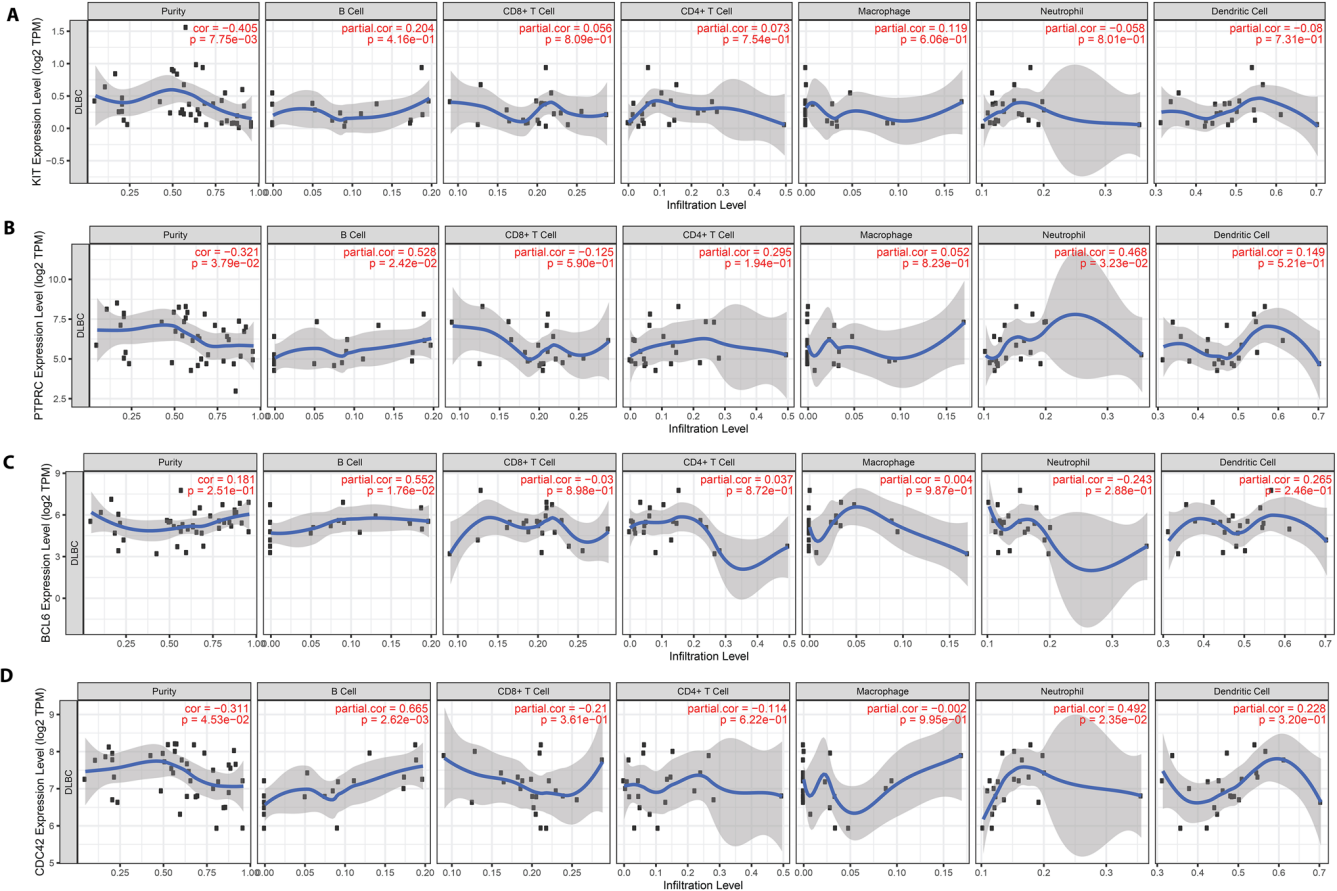
Correlation analysis of MTG1, PPP2R5B, and ANXA5 mRNA expression with blood cancer (lymphoma)-infiltrating immune cells. The data are from the TIMER database (https://cistrome.shinyapps.io/timer/). **A** Correlation of KIT mRNA with blood cancer-infiltrating immune cells. **B** Correlation of BCL6 mRNA with blood cancer-infiltrating immune cells. **C** Correlation of PTPRC mRNA with blood cancer-infiltrating immune cells. **D** Correlation of CDC42 mRNA with blood cancer-infiltrating immune cells

We imported the PPI network into Cytoscape to detect significant clustering modules. Module analysis and modules with top high scores were screened out (Fig. 6B). Eight hub nodes were present in the six modules (Table 2). According to GO and KEGG pathway enrichment analysis (*p* < 0.05), BCL6 in module 5 (MCODE score = 3.2) was closely associated with negative control of transcription from RNA polymerase II promoter, negative management of immune response, and negative regulation of the Notch signaling pathway. KIT in module 6 (MCODE score = 3.2) was involved in T-cell differentiation, mast cell degranulation, the RAS signaling pathway, MAPK cascade regulation of cell proliferation protease, and positive regulation of GTPase activity. PTPRC in module 3 (MCODE score = 5.2) was closely associated with antigen binding. EP300 was associated with the Jak-STAT signaling pathway, HTLV-I infection, transcriptional dysregulation in cancer, Influenza A, the FoxO signaling pathway, and melanogenesis.

**Table 2:** Hub gene module information

Hub	Module	MCODE score
EP300	Cluster Module 6	3.2
PTPRC	Cluster Module 3	5.2
KIT	Cluster Module 6	3.2
BCL6	Cluster Module 5	3.2
HNRNPC	Cluster Module 1	7.917
HNRNPA1	Cluster Module 1	7.917
GART	Cluster Module 2	7.818
SMAD	Cluster Module 13	2.667

### Molecular docking

**Fig. 7:**
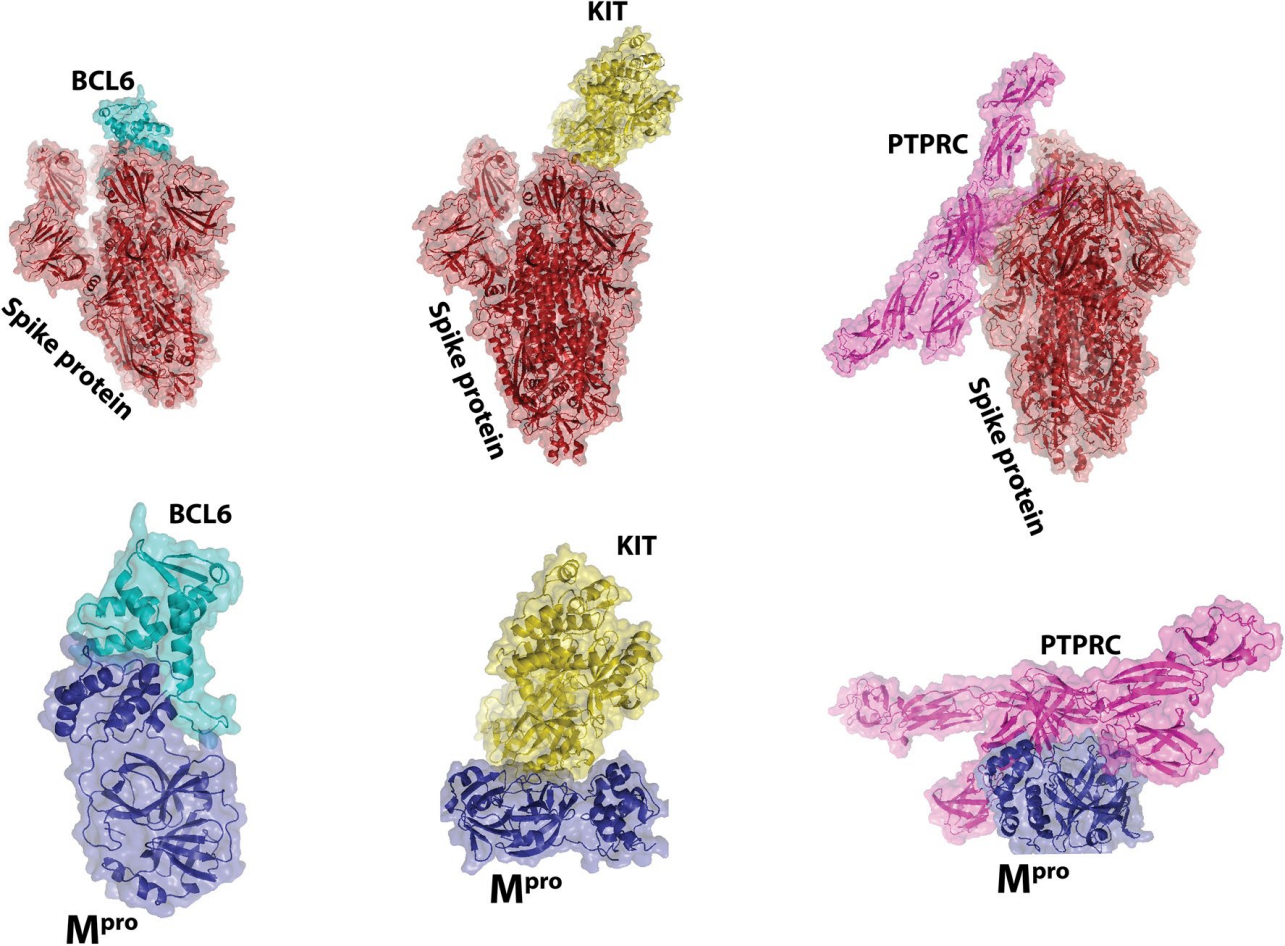
Molecular docking interactions and orientations for three common hub proteins in leukemia, and SARS-CoV-2 spike protein and Mpro. Docking interactions of **A** BCL6, **B** KIT, and **C** PTPRC. Visualization of interactions of these protein drug molecules with spike protein and Mpro, through PyMOL

**Fig. 8:**
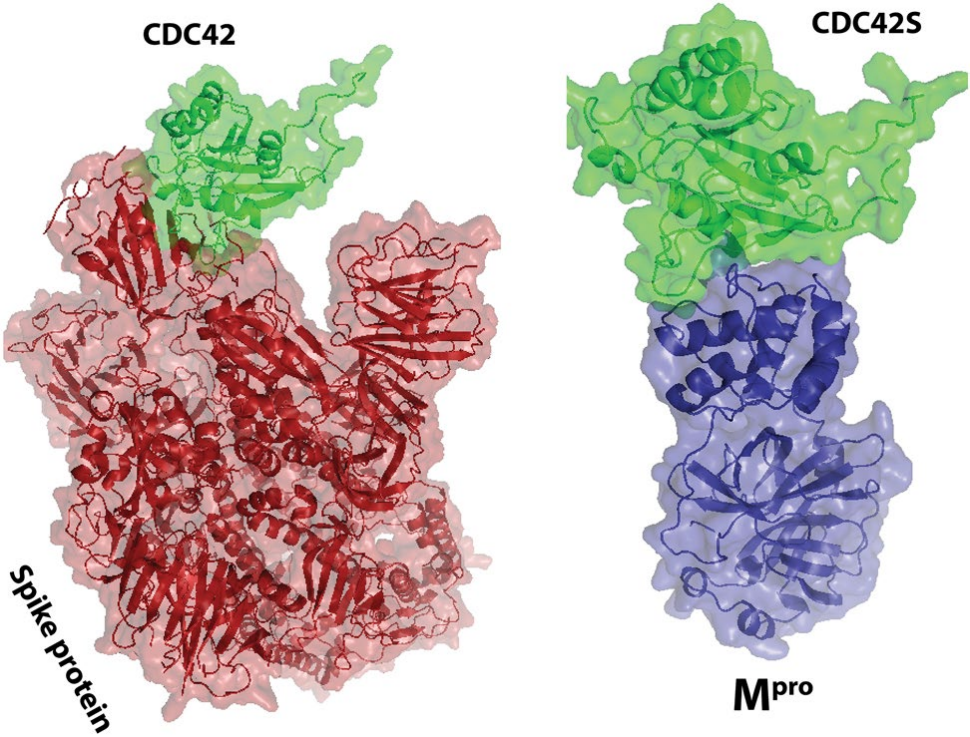
Molecular docking interactions and orientations of three hub proteins in leukemia with SARS-CoV-2 spike protein and Mpro. Docking interactions of **A** CDC42. Visualization of interactions of these protein drug molecules with spike protein and Mpro, through PyMOL

Only BCL6, KIT, and PTPRC were identified as up-regulated proteins common among the ten hub genes and 27 genes common in leukemia and SARS-CoV-2-associated proteins. The molecular docking of BCL6, KIT, and PTPRC with SARS-CoV-2 spike glycoprotein and Mpro is shown in Fig. 7. The Δ*G* of BCL6 and the spike protein of SARS-CoV-2 was − 56.7 kcal/mol, and the Δ*G* of BCL6 and the Mpro of the virus was − 6.8 kcal/mol. The $\mathrm{\Delta G}$ of KIT and the spike glycoprotein was − 52.5 kcal/mol, and the Δ*G* of KIT and the Mpro of the virus was − 12.3 kcal/mol. Finally, the Δ*G* of PTPRC and the spike glycoprotein was − 52.5 kcal/mol, and the Δ*G* of PTPRC and the Mpro of the virus was − 16.1 kcal/mol. The reported Δ*G* indicates the solvation free energy gain after interface formation; a negative Δ*G* indicates hydrophobic interfaces or positive protein affinity. The dissociation constant (*K*_d_) for each docking is given in Table 3. The three up-regulated proteins in the CLL and CML exhibited good interaction with the SARS-CoV-2 spike glycoprotein and Mpro, thus indicating that the blood tissues of patients with leukemia are vulnerable to SARS-CoV-2. The molecular docking for CDC42 (PDBID 1AJE) and SARS-CoV-2 spike glycoprotein, and CDC42 and Mpro is shown in Fig. 8. The Δ*G* of CDC42 and the spike glycoprotein was − 5.6 kcal/mol, and the Δ*G* of KIT and the Mpro of the virus was − 43.6 kcal/mol (Table 3).

**Table 3:** Docking results of the hub proteins of leukemia and the key proteins of SARS-CoV-2

Target protein involved in patients with leukemia	Virus protein	Δ*G* (kcal ^−1^ mol^−^1)	*K*_d_ (M) at 25 °C
CDC42	Main protease (Mpro)	− 5.6	8.50E−05
CDC42	Spike protein	− 43.6	1.00E−32
BCL6	Main protease (Mpro)	− 6.8	1.00E−05
BCL6	Spike protein	− 56.7	2.80E−42
PTPRC	Main protease (Mpro)	− 16.1	1.60E−12
PTPRC	Spike protein	− 49.9	2.40E−37
KIT	Main protease (Mpro)	− 12.3	8.80E−10
KIT	Spike protein	− 52.5	3.10E−39

## Discussion

In this study, we selected the spike glycoprotein and Mpro as the SARS-CoV-2 target proteins by using molecular docking technologies. Through the analysis of microarray datasets, we detected 385 overlapping DEGs. Enrichment analysis revealed 500 overlapping DEGs mainly associated with genes involved in the interferon response, type I interferon cellular response, virus response, viral genome replication regulation, immune effector process regulation, differentiation of T cells, activation of B cells, and signaling pathways of immune response-regulating cell surface receptor. Through molecular analysis, we selected ten hub genes from these 500 overlapping DEGs. The MAPK cascade, GTPase regulatory activity, and other factors listed herein were associated with leukemia. Subsequently, we identified only 27 leukemia-regulated SARS-CoV-2-associated proteins. Of the ten hubs, three genes (KIT, PTPRC, and BCL6) were common to both leukemia and SARS-Cov-2-associated proteins, which have been further studied in patients with COVID-19. CDC42 in human immunodeficiency virus (HIV)-1 cell entry is the most examined aspect of CDC42 function in viral cell entry processes [41]. Prior studies have demonstrated the role of CDC42 in cell entry of other RNA viruses—a critical area for further research through similar methods.

CDC42, a protein-coding gene, is involved in pathways including nerve growth factor (NGF) and the integrin pathway. Annotations associated with this gene in GO include similar protein binding and protein kinase binding. Furthermore, RAC1 is an essential paralog of this gene. In our study, CDC42 was the top hub (degree = 41). In addition, CDC42 has a crucial role in the entry process of mouse hepatitis coronavirus (MHV CoV) [42]. In the initial phases of infection, MHV infectivity and the use of actin cytoskeleton modifying agents had related restrictive events on infection, directing to GTPase, and explicitly to the participation of CDC42 in the entry process [41, 43]. Ethyl isopropyl amiloride (EIPA) is well known for its ability to inhibit macropinocytosis through inhibiting CDC42 signaling. The findings of diminished infectivity due to Arp2/3 knockdown, disruption of the actin cytoskeleton, and EIPA have indicated that CDC42 signaling is involved in the MHV cell entry process. Because the CDC42 hub gene is a crucial protein in leukemia, we performed molecular docking between CDC42 protein in leukemia (in CLL and CML) tissues and the spike glycoprotein and Mpro, the essential structural proteins of the SARS-CoV-2 virus. The hub proteins in leukemia successfully docked with the virus's essential proteins, thereby confirming our hypothesis that patients with leukemia can have a more significant threat of being attacked by SARS-CoV-2.

Clinical studies have shown that people of all ages are generally susceptible to COVID-19. By contrast, the risk of infection with the virus increases in older people and people with underlying diseases [44]. Management of patients with leukemia in the COVID-19 pandemic can be complicated. The risk of infection with SAR-Cov-2 remains low during high-risk COVID-19 periods when optimal preventive measures and mass testing are used; however, mortality may be elevated in patients with both leukemia and COVID-19. The effects of the COVID-19 pandemic on leukemia have been evaluated in recent reports, including a study of the incidence of anxiety in patients with leukemia during the COVID-19 pandemic [14, 18], a description of physical movement and quality lifestyle in patients with leukemia during the COVID-19 pandemic [14, 18], and an investigation of the outcomes of patients with leukemia affected by COVID-19 [18]. Recent research has shown that spike glycoprotein and Mpro [45] are the main structural proteins of COVID-19. Spike glycoprotein is the main target for COVID-19 vaccines, therapeutic antibodies, and diagnostics of COVID-19 [46]. Likewise, another possible target protein is Mpro (also called 3C-like protease), a key coronavirus enzyme with an essential role in facilitating viral replication and transcription, thus providing a promising drug target for COVID-19 [46].

A higher risk of infection and likelihood of severe COVID-19 was established among cancer patients as a sub-group early in the pandemic. Thus, this research provides valuable knowledge that should help physicians make informed choices in protecting and caring for patients with leukemia and COVID-19. Furthermore, this study lays a groundwork for future relevant laboratory studies, which may enable identification of novel potential molecular targets that may be exploited to inhibit viral interactions with host cellular factors and block the spread and viral replication in the body. A better structural understanding of molecular targets, virus–host interactions, and the cause of pathogenesis is required for the development of effective therapeutic/prophylactic agents for COVID-19 prevention and treatment.

## Conclusion

The findings of this bioinformatics-based research demonstrated that patients with chronic leukemia are at higher risk of SARS-CoV-2 infection as compared to normal individuals. Accordingly, patients with chronic leukemia require better medical attention during the COVID-19 pandemic.

## Supplementary Information

Below is the link to the electronic supplementary material.

Supplementary file1 Information on the up-regulated DEGs identified by GEO2R analysis of three GEO datasets (p-value < 0.05, |logFC|≥ 0.2 DEGs from GSE8835, GSE24739, and GSE39411. (XLSX 29 KB)

Supplementary file2 Information on for the downUP-regulated DEGs identified by the GEO2R analysis of three GEO datasets (p-value < 0.05, |logFC|≤ − 0.2 DEGs from GSE8835, GSE24739, and GSE39411. (XLSX 39 KB)

Supplementary file3 Gene enrichment analysis of up-regulated DEGs from chronic lymphocytic and chronic myelogenous samples of leukemia (XLSX 29 KB)

Supplementary file4 Gene enrichment analysis of down-regulated DEGs from chronic lymphocytic and chronic myelogenous samples of leukemia (XLSX 31 KB)

## Data Availability

The authors confirm that the data supporting the findings of this study are available within the article and its supplementary materials.
